# Handgrip and Pinch Grip Strength as Functional Indicators of Pediatric Malnutrition and Early Response to Nutritional Therapy: A Preliminary Single-Center Study

**DOI:** 10.3390/children13040531

**Published:** 2026-04-11

**Authors:** Mehmet Emin Yıldız, Tuğba Gürsoy Koca, Halil Kocamaz

**Affiliations:** 1 Department of Pediatrics, Ministry of Health Serinhisar District State Hospital, Denizli 20430, Turkey; mehmetemin.yildiz@saglik.gov.tr; 2 Department of Pediatrics, Division of Pediatric Gastroenterology, Hepatology and Nutrition, Pamukkale University Faculty of Medicine, Denizli 20070, Turkey; 3 Department of Pediatrics, Division of Pediatric Gastroenterology, Hepatology and Nutrition, Dicle University Faculty of Medicine, Diyarbakır 21280, Turkey; halil.kocamaz@dicle.edu.tr

**Keywords:** handgrip strength, pediatric malnutrition, muscle function, nutritional therapy, pinch grip strength

## Abstract

**Highlights:**

**What are the main findings?**

• Children with malnutrition have significantly lower handgrip and pinch grip strength compared with healthy peers, indicating reduced muscle mass and functional impairment.
• Handgrip strength shows strong positive associations with age, body weight, lean body mass, and mid-upper arm circumference, supporting its validity as a practical marker of nutritional status.
• After 8 weeks of individualized nutritional therapy, both anthropometric parameters and absolute muscle strength improve significantly.

**What are the implications of the main findings?**

• Handgrip and pinch grip strength measurements can serve as simple, non-invasive, and objective tools to complement routine anthropometric assessment in pediatric malnutrition.
• These functional measurements may help clinicians monitor early treatment response and guide nutritional management in daily practice.

**Abstract:**

**Background/Objectives:** Pediatric malnutrition is associated with loss of muscle mass and impaired physical function. While anthropometric measurements are widely used for diagnosis, functional indicators that reflect early changes in nutritional status are limited in children. Handgrip strength has been proposed as a simple and objective marker of muscle function; however, pediatric data remain scarce. **Methods**: In this prospective controlled study, 55 children aged 3–17 years diagnosed with malnutrition and 50 age- and sex-matched healthy controls were evaluated. Anthropometric measurements and muscle strength assessments, including handgrip and pinch grip strength, were performed in both groups. Muscle strength values were additionally converted to age- and sex-adjusted standard deviation scores (SDS). In the malnutrition group, measurements were repeated at 2 and 8 weeks following individualized nutritional therapy to assess treatment response. **Results**: Children with malnutrition had significantly lower body weight, body mass index, mid-upper arm circumference, triceps skinfold thickness, and lean body mass compared with controls (*p* < 0.05 for all). Both dominant and non-dominant handgrip strength values were also significantly reduced in the malnutrition group. When adjusted for age and sex, handgrip strength SDS values remained significantly lower in children with malnutrition, whereas pinch grip strength SDS values did not differ significantly between groups. During follow-up, nutritional therapy was associated with significant improvements in anthropometric parameters and absolute muscle strength measurements. However, SDS-based analyses demonstrated that these changes were not uniform across all parameters, suggesting that observed improvements may only partly exceed expected physiological growth. **Conclusions**: Handgrip strength appears to reflect nutritional status in children, and its association with malnutrition persists after adjustment for growth-related factors. These findings support its potential role as a complementary functional marker. However, longitudinal changes in standardized scores indicate that recovery is variable, and interpretation should consider the influence of normal growth and development. Further large-scale, age-standardized studies are needed to better define their role in clinical practice.

## 1. Introduction

Malnutrition is a nutritional disorder associated with adverse effects on body composition and physiological function [1,2,3,4,5]. In undernutrition, reductions in total body protein and cellular mass occur due to increased proteolysis and impaired protein synthesis. These metabolic changes are accompanied by diminished mitochondrial activity, leading to impaired muscle function and progressive loss of muscle mass and strength [3].

Muscle function is closely related to total body lean body mass, body cell mass, and anthropometric indicators such as mid-upper arm circumference (MUAC) and body mass index (BMI). Accordingly, weight loss is often accompanied by reduced muscle strength. Handgrip and pinch grip strength measurements have been widely used in adult populations as reliable indicators of muscle function and nutritional status, both in healthy individuals and in those with malnutrition or chronic disease. While handgrip strength (HGS) reflects global muscle function, pinch grip strength (PGS) may provide additional information regarding fine motor strength and intrinsic hand muscle function. These smaller muscle groups may be affected earlier in conditions associated with protein-energy deficiency. Therefore, evaluating both HGS and PGS may offer a more comprehensive assessment of functional impairment in pediatric malnutrition. Several studies have demonstrated that these functional indices correlate strongly with lean body mass, physical performance, and clinical outcomes in adults [6,7]. However, despite this well-established role in adult populations, data regarding their utility in pediatric patients remain limited. This gap highlights the need to evaluate whether these simple, non-invasive functional measures can be applied effectively in children, particularly for the early detection of malnutrition and monitoring of treatment response.

Pediatric malnutrition adversely affects growth and development due to a sustained imbalance between nutritional intake and physiological requirements [5]. Compared with adults, children are more vulnerable to its consequences, particularly during critical periods of growth, making early identification essential for improving outcomes and reducing morbidity [8]. However, screening remains challenging in outpatient and community settings, as anthropometric measures may not fully capture functional impairment, and no universally accepted screening tool exists for the general pediatric population [4,9]. In addition, muscle strength in pediatric populations is strongly influenced by age, growth, and somatic development, which complicates the interpretation of raw strength measurements across a wide age range.

In this context, functional assessments such as handgrip and pinch grip strength may provide complementary information beyond conventional measurements. The present study was designed as a preliminary investigation to explore the potential clinical utility of muscle strength measurements in pediatric malnutrition. We hypothesized that HGS and PGS would be significantly reduced in children with malnutrition and would demonstrate measurable responsiveness to short-term nutritional rehabilitation, thereby supporting their potential utility as functional indicators of both disease severity and treatment response.

## 2. Materials and Methods

### 2.1. Study Design and Population

This study was designed as a preliminary, single-center, prospective observational study with a control group, conducted at the Pediatric Gastroenterology and General Pediatrics outpatient clinics of Pamukkale University. Between 1 January 2022 and 1 August 2022, a total of 55 children aged 3–17 years who presented with various complaints and were diagnosed with malnutrition at initial evaluation were consecutively enrolled. The control group comprised 50 age- and sex-matched healthy children with normal nutritional status (BMI SDS ≥ −2), no history of acute or chronic systemic disease, and no acute illness at the time of assessment.

Inclusion criteria for the malnutrition group were: (i) age between 3 and 17 years, (ii) diagnosis of malnutrition based on WHO criteria, and (iii) ability to comply with measurement procedures. Exclusion criteria included: (i) presence of orthopedic or neurological conditions affecting upper extremity function, (ii) inability to understand or follow verbal instructions, and (iii) any chronic systemic disease that could affect nutritional status or muscle function.

### 2.2. Data Collection and Measurements

Demographic data were recorded for all participants, and anthropometric measurements, including height, body weight, BMI, weight-for-height, mid-upper arm circumference, and triceps skinfold thickness, were obtained using standardized techniques in accordance with established protocols [10]. Height was measured to the nearest 0.1 cm using a Veeder-Root stadiometer (Elizabethtown, NC, USA), with participants standing barefoot in an upright position. Body weight was measured to the nearest 0.1 kg using a calibrated electronic scale (Seca GmbH & Co. KG, Hamburg, Germany), with participants wearing light clothing. Body composition parameters, including body fat percentage and lean body mass, were assessed using bioelectrical impedance analysis with a Tanita BC-601 body composition analyzer (Tanita Corporation, Tokyo, Japan) under standardized conditions. All anthropometric, body composition, and muscle strength measurements were performed by the same trained healthcare professional to minimize inter-observer variability. HGS was assessed using a Baseline BIMS Digital Hand Dynamometer (Fabrication Enterprises Inc., White Plains, NY, USA) following the National Health and Nutrition Examination Survey (NHANES) protocol [6,11]. Measurements were performed in the seated position with the elbow flexed at 90°. Participants were instructed to apply maximal force and maintain the grip for 5 s. Three measurements were obtained for each hand (dominant and non-dominant) with 60 s rest intervals, and the highest value was recorded. Hand dominance was determined based on the preferred hand used for daily activities, as reported by the child or caregiver, particularly in younger children where dominance may not be fully established. PGS was measured using a Baseline Electronic Pinchmeter (Fabrication Enterprises Inc., White Plains, NY, USA) without changing the participant’s position. Lateral pinch (key grip) technique was used, with the thumb placed on the upper arm and the index finger on the lower arm of the device. Measurements were repeated three times for each hand with 60 s rest intervals, and mean values were recorded in kilograms [11]. For HGS, the highest value was recorded in accordance with standard protocols such as NHANES, reflecting maximal voluntary muscle strength. For PGS, mean values were used to reduce measurement variability and improve reliability of fine motor strength assessment.

#### Z-Score Calculation for Muscle Strength

Handgrip strength and PGS values were standardized to account for age- and sex-related differences in growth and muscle development in the pediatric population.

For HGS, two different approaches were used depending on age. In preschool children (3–6 years), where continuous age-specific LMS parameters are limited, Z-scores were calculated using age- and sex-specific normative mean and standard deviation (SD) values reported in the literature [12]. Z-scores were derived using the standard formula: Z = (X − mean)/SD, where X represents the measured HGS value (kg), and mean and SD correspond to the appropriate reference values. When age was not an integer, reference values were estimated by interpolation between adjacent age points. For children aged 6–17 years, Z-scores were calculated using the LMS (Lambda, Mu, Sigma) method, which accounts for skewness in the distribution and is widely accepted for pediatric reference modeling [13]. Age-, sex-, and hand dominance-specific reference parameters were obtained from the NHANES 2011–2014 dataset. Individual Z-scores were calculated using the formula Z = [(X/M)^L^ − 1]/(L × S); when L = 0, the formula Z = ln(X/M)/S was applied.

Pinch grip strength values were standardized separately using published pediatric normative data stratified by age, sex, and hand dominance [14]. Z-scores were calculated using the standard formula: Z = (X − μ)/σ, where X represents the observed PGS value (kg), μ the reference mean, and σ the reference standard deviation. When necessary, reference values were obtained by interpolation between adjacent age points.

The use of different Z-score calculation methods reflects the structure of the available reference datasets. The standard Z-score formula was applied when only summary statistics (mean and SD) were available, whereas the LMS method was used when distribution parameters (L, M, S) were provided.

Z-scores were calculated separately for dominant and non-dominant hands.

All measurements were performed by the same trained healthcare professional to ensure consistency. Standardized measurement procedures based on the NHANES protocol were followed to minimize intra-observer variability. Although formal intra-class correlation coefficient (ICC) analysis was not performed, handgrip and pinch grip strength measurements have been previously validated in pediatric populations, including young children, when conducted using standardized techniques and appropriate equipment. Measurements were performed under standardized conditions during outpatient visits, and participants were instructed to perform maximal effort. No strict timing in relation to meals was applied; however, all measurements were conducted under similar clinical conditions to reduce variability.

Relative handgrip strength (rHGS) was calculated by dividing the maximum dominant HGS by BMI (kg/kg/m^2^) [15]. Body mass index (BMI, Quetelet index) was calculated for descriptive purposes. However, nutritional status classification and statistical comparisons were primarily based on BMI-for-age z scores (BMI SDS), in accordance with WHO recommendations for pediatric populations.

Anthropometric parameters were expressed as age- and sex-adjusted z scores calculated according to the World Health Organization (WHO) growth standards using the WHO Anthro (v3.2.2) and AnthroPlus software (v1.0.4) [16]. In children younger than 5 years, nutritional status was evaluated using weight-for-height (WFH) and height-for-age (HFA) z scores, whereas BMI-for-age and HFA z scores were applied in children aged 5 years and older, in accordance with WHO and European Society for Pediatric Gastroenterology, Hepatology and Nutrition (ESPGHAN) recommendations [1,5]. Nutritional status was classified based on WHO criteria, with z scores ≥ −2 standard deviation score (SDS) considered normal, values between −2 and −3 SDS indicating moderate malnutrition, and z scores < −3 SDS defining severe malnutrition. BMI SDS, which expresses BMI relative to age- and sex-specific reference populations, was used to allow standardized comparison across pediatric age groups.

Laboratory parameters were assessed as part of routine clinical evaluation but were not included in the primary analysis of this study.

### 2.3. Treatment and Follow-Up

Following baseline evaluation, children diagnosed with malnutrition were provided with individualized nutritional therapy tailored to their ideal body weight and age-specific daily energy requirements. Anthropometric parameters as well as HGS and PGS measurements were reassessed at the second and eighth weeks of follow-up. To evaluate the true effect of the intervention and to minimize the potential confounding effect of physiological growth during this period, Z-scores were calculated separately for each visit using the participants’ updated decimal age. Laboratory evaluations were repeated at the eighth week to assess biochemical changes associated with nutritional recovery.

### 2.4. Statistical Analysis

Statistical analyses were performed using IBM SPSS Statistics for Windows, version 25.0 (IBM Corp., Armonk, NY, USA). The *t*-test was used for data conforming to a normal distribution, and the Mann–Whitney U test was used for those that did not. Analysis of variance or Friedman’s test was used for repeated measures analysis. A *p* value < 0.05 was considered statistically significant. Normality of data distribution was assessed using the Shapiro–Wilk test. Continuous variables are presented as mean ± standard deviation. Group comparisons for SDS values were performed using independent samples *t*-tests, while longitudinal changes were assessed using paired samples *t*-tests.

### 2.5. Ethical Approval

Ethical approval for the study was obtained from the Pamukkale University Non-Interventional Clinical Research Ethics Committee (approval number: 13; 13 July 2021). This study was conducted as a single-center study at Pamukkale University. All study procedures, including patient recruitment, data collection, and follow-up assessments, were performed exclusively at Pamukkale University. At the time of the study, all contributing authors were affiliated with this institution. The current institutional affiliations of the co-authors reflect their positions at the time of manuscript submission and do not indicate multicenter involvement.

The study was conducted in accordance with the Helsinki Declaration.

## 3. Results

### 3.1. Demographic Characteristics and Anthropometry

A total of 55 children with malnutrition and 50 healthy controls were included in the baseline analysis. Follow-up data were available for 45 patients at the second visit (approximately 2 weeks) and for 36 patients at the final (8-week) assessment. The number of participants included in each analysis varied depending on data availability. There were no statistically significant differences between the malnutrition and control groups in terms of age (11.32 ± 4.03 vs. 10.54 ± 3.89 years, *p* = 0.315) or sex distribution (female: 56.4% vs. 62.0%, *p* = 0.698), confirming appropriate matching between groups. Based on WHO z-score classification, the study cohort included children with both moderate (−2 to −3 SDS) and severe (<−3 SDS) malnutrition. Although the exact distribution was not separately analyzed, the mean BMI SDS value (−2.33 ± 1.01) suggests that the cohort predominantly consisted of moderately malnourished children, with a subset of more severely affected cases. Comparative analyses of anthropometric parameters are presented in Table 1. Children with malnutrition had significantly lower body weight, BMI, and BMI SDS compared with healthy controls (all *p* < 0.001). BMI SDS showed significant differences between groups. In addition, lean body mass was markedly reduced in the malnutrition group (*p* = 0.001). Mid-upper arm circumference and triceps skinfold thickness (TSF) measurements were also significantly lower among malnourished children (both *p* < 0.0001).

### 3.2. Muscle Strength Measurements

Comparisons of muscle strength parameters between the malnutrition and control groups are summarized in Table 1. Children with malnutrition demonstrated significantly lower HGS in both the dominant and non-dominant hands compared with healthy controls. Maximum HGS was also significantly reduced in the malnutrition group. No significant difference was observed between the malnutrition and control groups in dominant hand PGS (3.62 ± 1.60 vs. 4.15 ± 1.49 kg, *p* = 0.082). When analyzed using age- and sex-adjusted standardized scores, dominant HGS SDS was significantly lower in the malnutrition group compared to controls (−2.64 ± 4.87 vs. −0.61 ± 1.10, *p* = 0.004). Similarly, non-dominant HGS SDS was significantly reduced (−2.13 ± 2.87 vs. −0.41 ± 1.16, *p* < 0.001). In contrast, no significant differences were observed in PGS SDS values between groups for either the dominant or non-dominant hand (*p* > 0.05). With respect to PGS, malnourished children showed significantly lower values in the non-dominant hand, whereas no significant difference was observed for the dominant hand. The number of participants included in each analysis varied according to the availability of specific anthropometric and body composition measurements. These findings indicate that reduced HGS in malnourished children persists even after adjustment for age and sex, whereas PGS appears less affected when standardized for growth-related factors.

### 3.3. Assessment of Treatment Response

Patients with malnutrition who initiated nutritional therapy underwent follow-up assessments at a mean of 16.8 ± 5.4 days for the second evaluation and 80.6 ± 33.9 days for the third evaluation after baseline. Follow-up data were available for 45 patients at the second visit and for 36 patients at the third visit. At the early follow-up (approximately two weeks), data were available for 45 patients. A modest increase was observed in body weight and muscle strength parameters compared with baseline; however, these changes were generally small and did not reach statistical significance for most variables. Analyses demonstrated significant increases in height, body weight, BMI, and BMI SDS compared with baseline values (all *p* < 0.001).

When evaluated using standardized scores, the increase in HGS SDS over time did not reach statistical significance (*p* = 0.214), suggesting that part of the observed improvement in absolute muscle strength may be attributable to normal growth rather than purely treatment-related effects.

Handgrip strength increased significantly in both the dominant and non-dominant hands during follow-up, with the greatest improvement observed at the final measurement (Table 2). Pinch grip strength also showed significant gains in both hands. In contrast, BMI-adjusted rHGS remained largely unchanged throughout the study period. Overall, absolute muscle strength improved alongside anthropometric recovery during nutritional treatment. In contrast, rHGS did not differ significantly between groups and did not show significant change during follow-up. Effect size analysis demonstrated small-to-moderate improvements, with the largest effect observed for BMI SDS (d = 0.45) and dominant hand PGS (d = 0.32). When analyzed using standardized scores, non-dominant HGS SDS and dominant PGS SDS showed significant improvement over time (*p* = 0.004 and *p* = 0.017, respectively), whereas dominant HGS SDS and non-dominant PGS SDS did not demonstrate significant change.

Laboratory follow-up revealed a significant increase in mean hemoglobin, mean corpuscular volume, total iron-binding capacity, and folate levels, accompanied by significant reductions in platelet count, ferritin, homocysteine, and retinol-binding protein concentrations. No significant changes were detected in the remaining laboratory parameters.

Correlation analyses were performed across the entire study population covering the full pediatric age range. Correlation analyses demonstrated strong positive associations between mean dominant HGS and age, body weight, height, lean body mass, and MUAC (r = 0.920, 0.947, 0.919, 0.934, and 0.895, respectively). HGS also showed a moderate positive correlation with BMI and body fat mass (r = 0.932 and 0.606, respectively). In contrast, the association between HGS and TSF was weak but remained positive (r = 0.283).

## 4. Discussion

Pediatric malnutrition continues to represent a substantial global health problem, with important implications for growth, development, and long-term functional outcomes. In clinical practice, early identification of malnutrition remains challenging, particularly in outpatient settings, where screening tools must be rapid, reliable, and easily applicable. Beyond conventional anthropometric measurements, functional indicators that reflect muscle performance may offer additional insight into the physiological impact of nutritional deficiency. In this context, the present study evaluated handgrip and pinch grip strength as functional indicators of malnutrition in children and examined their response to nutritional therapy.

Consistent with previous reports, muscle strength measurements in our cohort increased with age and showed strong positive correlations with age-related growth parameters [17,18,19]. HGS demonstrated particularly high correlations with body weight, height, and lean body mass, supporting the close relationship between muscle function, somatic growth, and total body lean mass [18,20,21]. These findings are in line with earlier pediatric studies showing that muscle strength reflects not only muscle mass but also overall nutritional and developmental status [22,23]. The absence of a significant difference between boys and girls further supports existing evidence that sex-related differences in muscle strength are limited in childhood, especially before late pubertal stages [18].

As expected, children with malnutrition exhibited significantly lower anthropometric indices, including BMI, BMI-SDS, MUAC, and TSF, compared with healthy controls. Importantly, these structural deficits were accompanied by clear reductions in both handgrip and pinch grip strength, indicating that malnutrition affects not only body composition but also functional capacity. Previous studies have highlighted HGS as a reliable surrogate for muscle mass and as a correlate of MUAC [24]. Our findings reinforce this association and extend it by demonstrating similar patterns for PGS, particularly in the non-dominant hand, suggesting a generalized impairment in muscle performance rather than a task-specific or hand-dominance-related deficit. A key strength of the present study is the use of standardized deviation scores to account for growth-related confounding. In pediatric populations, muscle strength is strongly influenced by age, sex, and somatic development, making interpretation of raw values challenging. Our SDS-based analyses demonstrated that HGS remains significantly reduced in children with malnutrition even after adjustment for these factors, indicating impaired functional status relative to age-specific expectations. In contrast, PGS did not differ significantly between groups when analyzed using SDS, suggesting that fine motor strength may be relatively preserved compared to global muscle function in pediatric malnutrition. The heterogeneous pattern observed in SDS changes suggests that short-term nutritional therapy may lead to selective improvements in muscle function rather than uniform recovery across all strength parameters.

Laboratory markers are frequently used in the evaluation of nutritional status; however, their interpretation is often complicated by the influence of inflammation and acute illness. Nevertheless, these parameters alone may not fully capture early or functional changes in nutritional status [25]. In contrast, muscle strength measurements appeared to reflect nutritional impairment more directly and showed measurable improvement during follow-up. This observation supports the notion that functional assessments may provide additional insight into changes during recovery than selected biochemical markers [3].

One of the notable findings of this study is the significant improvement in both anthropometric measures and muscle strength parameters following nutritional therapy. Handgrip and pinch grip strength increased in parallel with gains in body weight, BMI SDS, and body composition indices. While improvements in HGS after nutritional support have been well documented in adult populations [6,26], pediatric data remain limited. Our results contribute to the growing evidence that muscle strength measurements are responsive to nutritional intervention in children and may serve as complementary tools to support the assessment of treatment response in clinical practice [23]. The lack of significant change in BMI-adjusted rHGS suggests associations with increases in body size and lean body mass to increases in body size and lean body mass during recovery. Although statistically significant improvements were observed in several parameters, effect size analysis revealed that the magnitude of change was generally small to moderate. This suggests that short-term nutritional intervention leads to measurable but gradual functional recovery.

The wide age range of the study population (3–17 years) should be carefully considered when interpreting muscle strength measurements. In pediatric populations, handgrip and pinch grip strength are strongly influenced by age, growth, and pubertal development, reflecting increases in muscle mass, neuromuscular coordination, and overall somatic maturation. Therefore, the very strong correlations observed between HGS and anthropometric parameters such as height, body weight, and lean body mass are expected and likely reflect normal developmental processes rather than a disease-specific effect. This highlights the importance of considering age- and sex-related growth when interpreting raw muscle strength values. The use of standardized indices, such as age- and sex-adjusted z-scores, or statistical approaches controlling for developmental factors, may provide a more accurate assessment of the independent impact of malnutrition on muscle function. In the present study, such adjustments were not performed, which represents an important limitation and should be addressed in future research. Importantly, the use of SDS-based analysis demonstrates that some of the observed improvements exceed expected growth-related changes while others do not, highlighting the complexity of interpreting functional recovery in pediatric populations.

An important result of the present study is that rHGS, calculated as HGS adjusted for BMI, did not differ significantly between children with malnutrition and healthy controls and did not change significantly during follow-up. This observation suggests that the differences observed in absolute HGS are, at least in part, influenced by variations in body size and overall growth rather than reflecting an isolated impairment in muscle function. In other words, lower absolute muscle strength in malnourished children may largely parallel reduced body mass and lean tissue rather than indicating a disproportionate functional deficit. This finding highlights the importance of interpreting absolute muscle strength values in the context of body size and supports the use of standardized or size-adjusted indices in pediatric populations. Accordingly, while absolute handgrip and pinch grip strength measurements provide useful information, their interpretation should consider growth-related factors, and they may be most appropriately used as complementary indicators alongside anthropometric assessment. Although absolute muscle strength improved during follow-up, the lack of significant change in SDS values suggests that short-term improvements may partly reflect normal growth rather than true functional recovery. This highlights the importance of using age-adjusted measures when evaluating treatment response in growing children.

The present study has several limitations. First, the lack of stratification according to malnutrition severity limits the ability to assess differences between moderate and severe cases, where functional impairment may vary. In addition, the relatively wide age range of the study population (3–17 years) introduces substantial developmental variability, as muscle strength in children is strongly influenced by growth and maturation. This heterogeneity may have affected the strength of the observed associations and limits the ability to isolate the independent effect of malnutrition. Second, measurement reliability in very young children may be affected by cooperation and attention span, despite the use of standardized protocols. Third, the use of raw muscle strength values instead of age- and sex-adjusted standard deviation scores restricts the ability to interpret findings independently of growth-related factors. In addition, partial correlation analyses adjusting for age and sex were not performed, which may limit the assessment of the independent effect of malnutrition on muscle strength. Despite the use of SDS-based analysis, the relatively small sample size and limited follow-up duration may have reduced the ability to detect significant changes in standardized muscle strength over time. The number of participants decreased during follow-up, with fewer patients available at subsequent assessments, which may have introduced bias and reduced the statistical power of longitudinal analyses. The relatively modest sample size and the absence of repeated measurements in the control group further limit the evaluation of longitudinal changes. Finally, although several parameters showed statistically significant improvement during follow-up, the magnitude of change was generally small to moderate. This distinction is important, as statistical significance does not necessarily imply clinical relevance. Bioelectrical impedance analysis may also have limited sensitivity in younger children and can be influenced by hydration status. Despite these limitations, the use of standardized measurement protocols and the combined assessment of handgrip and pinch grip strength strengthen the reliability of the findings.

## 5. Conclusions

This study demonstrates that handgrip and pinch grip strength are reduced in children with malnutrition and improve following nutritional therapy. These functional measures correlate with anthropometric and body composition parameters and appear to reflect changes in nutritional status over time. Muscle strength assessments may offer a practical, rapid, and non-invasive approach to complement conventional anthropometric evaluation, particularly in outpatient settings where simple tools are needed. Age-adjusted analyses indicate that HGS is reduced relative to expected values in malnourished children, whereas improvements over time should be interpreted cautiously. Unlike some biochemical markers, which may be influenced by inflammation and may not reflect short-term changes, functional measures may provide additional insight into the recovery of muscle function. However, given the study design, relatively modest sample size, and wide age range of participants, these findings should be interpreted with caution. Muscle strength measurements should be considered as complementary rather than standalone indicators of nutritional status. Further large-scale, age-standardized studies are needed to validate their routine use in clinical practice.

## Figures and Tables

**Table 1:** Comparison of anthropometric, body composition, and muscle strength parameters between children with malnutrition and healthy controls.

Variable	Malnutrition Mean ± SD (n)	Control Mean ± SD (n)	*p* Value
Age (years)	11.32 ± 4.03	10.54 ± 3.89	0.315
Sex (F/M)	31/24	31/19	0.698
BMI (kg/m^2^)	14.58 ± 1.98 (55)	18.62 ± 3.48 (50)	<0.001
BMI SDS	−2.33 ± 1.01 (55)	0.02 ± 1.09 (50)	<0.001
Body fat mass (kg)	4.55 ± 1.71 (49)	8.89 ± 4.34 (44)	<0.001
Lean body mass (kg)	23.72 ± 9.29 (49)	30.69 ± 11.00 (44)	0.001
MUAC (cm)	18.1 ± 2.96 (54)	22.58 ± 3.79 (49)	<0.001
TSF (mm)	8.76 ± 3.43 (54)	16.88 ± 6.17 (49)	<0.001
HGS—maximum (kg)	14.45 ± 8.03 (55)	17.99 ± 8.93 (50)	0.047
rHGS	0.96 ± 0.44 (55)	0.95 ± 0.43 (50)	0.905
HGS—dominant (kg)	13.52 ± 7.71 (55)	16.92 ± 8.48 (50)	0.043
HGS—SDS (dominant)	−2.64 ± 4.87 (55)	−0.61 ± 1.10 (50)	0.004 *
HGS—non-dominant (kg)	12.51 ± 7.11 (55)	16.75 ± 8.62 (50)	0.011
HGS—SDS (non-dominant)	−2.13 ± 2.87 (55)	−0.41 ± 1.16 (50)	<0.001 *
PGS—dominant (kg)	3.62 ± 1.60 (55)	4.15 ± 1.49 (50)	0.082
PGS—SDS (dominant)	0.33 ± 4.16 (55)	0.89 ± 1.68 (50)	0.361 *
PGS—non-dominant (kg)	3.26 ± 1.49 (55)	3.96 ± 1.56 (50)	0.019
PGS—SDS (non-dominant)	0.38 ± 4.37 (55)	0.88 ± 1.69 (50)	0.433 *

BMI: body mass index, HGS: handgrip strength, rHGS: relative handgrip strength, MUAC: mid-upper arm circumference, PGS: pinch grip strength, SD: standard deviation, TSF: triceps skinfold thickness, F: female, M: male, SDS: standard deviation score (age- and sex-adjusted values). * Independent samples *t*-test (Welch correction when appropriate).

**Table 2:** Changes in anthropometric, body composition and muscle strength parameters at 8 weeks after nutritional therapy in children with malnutrition (n: 36).

Variable	Baseline (Mean ± SD)	Week 8 (Mean ± SD)	*p* Value *
Height (cm)	136.2 ± 20.2	137.0 ± 19.8	<0.001
Body weight (kg)	28.6 ± 10.5	30.0 ± 10.6	<0.001
BMI SDS	−2.49 ± 0.86	−2.05 ± 1.07	<0.001
Body fat mass (kg)	4.9 ± 1.8	5.4 ± 2.0	0.001
Lean body mass (kg)	25.6 ± 9.4	26.6 ± 9.5	<0.001
MUAC (cm)	18.5 ± 3.0	19.3 ± 3.1	<0.001
rHGS	1.01 ± 0.44	1.03 ± 0.44	0.247
HGS—dominant (kg)	14.4 ± 7.9	15.4 ± 8.0	<0.001
HGS—SDS (dominant)	−2.83 ± 5.43	−1.95 ± 3.18	0.214 ^¥^
HGS—non-dominant (kg)	13.2 ± 7.2	14.1 ± 7.0	0.002
HGS—SDS (non-dominant)	−2.16 ± 2.95	−1.90 ± 2.87	0.004 ^¥^
PGS—dominant (kg)	3.8 ± 1.7	4.4 ± 2.0	<0.001
PGS—SDS (dominant)	0.34 ± 4.37	1.05 ± 4.98	0.017 ^¥^
PGS—non-dominant (kg)	3.5 ± 1.6	3.9 ± 1.7	<0.001
PGS—SDS (non-dominant)	0.51 ± 4.71	0.88 ± 4.74	0.213 ^¥^

BMI: body mass index, HGS: handgrip strength, rHGS: relative handgrip strength, MUAC: mid-upper arm circumference, PGS: pinch grip strength, SD: standard deviation, TSF: triceps skinfold thickness, SDS: standard deviation score (age- and sex-adjusted values). * *p* values derived from repeated-measures analysis (Friedman test or repeated-measures ANOVA, as appropriate). ^¥^ Paired samples *t*-test.

## Data Availability

The original contributions presented in this study are included in the article. Further inquiries can be directed to the corresponding author.

## Abbreviations

The following abbreviations are used in this manuscript:

BMI	Body mass index
ESPGHAN	European Society for Paediatric Gastroenterology, Hepatology and Nutrition
HFA	Height-for-age
HGS	Handgrip strength
IGF-1	Insulin-like growth factor 1
NHANES	National Health and Nutrition Examination Survey
PGS	Pinch grip strength
rHGS	Relative handgrip strength
RBP	Retinol-binding protein
SDS	Standard deviation score
TSF	Triceps skinfold thickness
WFH	Weight-for-height
WHO	World Health Organization
